# F144. MUSCARINIC M1 RECEPTOR SIGNALLING UNDERLYING COGNITION IN PSYCHOTIC DISORDERS

**DOI:** 10.1093/schbul/sby017.675

**Published:** 2018-04-01

**Authors:** Therese van Amelsvoort, Geor Bakker, Claudia Vingerhoets, Barbara J Sahakian, Oswald Bloemen, Matthan Caan, Jan Booij

**Affiliations:** 1 Maastricht University; 2 University of Cambridge; 3 GGz Centraal, Maastricht University; 4 Academic Medical Centre

## Abstract

**Background:**

Antipsychotic treatment has failed to improve cognitive deficits associated with psychotic disorders. This has led to an increased interest to revisit earlier implications from post-mortem studies that lowered muscarinic M1 receptor signaling may underlie these symptoms. This receptor is highly expressed in important regions for cognition such as the dorsolateral prefrontal cortex (DLPFC) and hippocampus, and administration of anti-muscarinic agents gives induce cognitive deficits in healthy volunteers. Administration of xanomeline, a M1/4 agonist in patients with schizophrenia resulted in improved learning and memory scores and decreased psychotic symptom severity. Therefore, the current study sought to examine alterations in muscarinic M1 receptor signaling in relation to cognitive functioning in medication free subjects with psychotic disorders and matched controls.

**Methods:**

Muscarinic M1 binding potential (BPND) was measured using single photon emission computed tomography (SPECT) with the M1 selective radiopharmaceutical 123I-iododexetimide in the DLPFC and hippocampus in the psychotic group. Pharmacological functional magnetic resonance imaging (phMRI) with the M1 antagonist biperiden was used to assess differences in functional response on the paired associate learning task (PAL) and emotion recognition task (ERT) adapted for fMRI from The Cambridge Neuropsychological Test Automated Battery in all subjects. The PAL task assessed encoding phase (learning) and retrieval (memory) of figure-place associations and the ERT task social cognition, both highly predictive of functional outcome. Cluster significance was set at Z>2.3, with cluster threshold correction at p<0.05.

**Results:**

The current study included 26 (mean age: 27.68; 19male/7 female) subjects with a psychotic disorder and 29 (mean age 25.63; 20 male/9 female) matched controls. Subjects with psychotic disorders recalled less figure place associations than controls (t=2.9, p=0.005) and were worse in recognizing different intensities of disgust emotions (t=2.26, p=0.03). Psychotic subjects showed a blunted response in functional reactivity to biperiden in the bilateral superior and medial frontal gyri with decreasing intensity of disgust facial expressions compared to controls, this blunted response was greatest in those with lower M1 BPND in the DLPFC. During encoding processes, psychotic subjects also showed differential reactivity to biperiden in the left middle frontal gyrus, insula, and caudate nucleus showing hypoactivation compared to controls. Greater hypoactivation was significantly associated with lower hippocampal M1 BPND. For retrieval both groups showed lowered activation under biperiden in the inferior frontal gyrus, but psychotic subjects failed to show increased activation with increasing cognitive load in the placebo condition, like the controls. Lower hippocampal M1 BPND in psychotic subjects was associated with lower activation of this region.

**Discussion:**

Results show preliminary evidence for altered M1 signaling of prefrontal areas in psychotic disorders underlying social cognition and learning and memory processes. Additionally, results show an important role for the M1 receptor in the DLPFC and hippocampus in altered fronto-striatal activation underlying encoding processes. Lower hippocampal M1 BPND is related to more severe alterations in underlying functional activation in encoding and retrieval processes. Results further support the need for development of therapeutic strategies that focus on the M1 receptor to improve cognitive functioning and functional outcome in psychosis.

